# Blakiston’s Quiz Compends

**Published:** 1904-01

**Authors:** 


Blakiston's Quiz Compends. A Compend of Diseases of the Skin. By Jay F. Schamberg, A. B., M. D., Professor of Diseases of the Skin, Philadelphia Polyclinic and College for Graduates in Medicine. Third edition, revised and enlarged, with 106 illustrations. Philadelphia: P. Blakiston's Son & Co. 1903. (Price, 80 cents.)

Five years ago the writer reviewed this manual, stating that it seemed to be a most creditable effort of the kind although, in general, such small works are not to be commended. What was good before has now been improved, through the labors of Dr. Schamberg, whose original investigations into various kinds of skin diseases are of so much consequence to the profession. The fact that the work has reached its fifth edition shows that it has met admirably the needs of the student and practitioner. Naturally it deals only with elementary facts of dermatology,—those most necessary for the student in schools where some knowledge of specialties is required. The photographic illustrations are about the same as in the former editions, although they seem to have been printed with more scrupulous care.

